# A Case Report of Cytomegalovirus-Associated Anterior Uveitis With Atypical Bilateral Circumferential Peripheral Iris Atrophy

**DOI:** 10.7759/cureus.94470

**Published:** 2025-10-13

**Authors:** Taisei Nagao, Kohei Morimatsu, Aika Tsutsui, Kazunobu Sugihara, Masaki Tanito

**Affiliations:** 1 Department of Ophthalmology, Shimane University Faculty of Medicine, Izumo, JPN

**Keywords:** anterior uveitis, cytomegalovirus, iris atrophy, secondary glaucoma, trabeculectomy, trabeculotomy

## Abstract

Cytomegalovirus-associated anterior uveitis (CMV-AU) is an important subtype of herpesvirus anterior uveitis, usually unilateral and characterized by recurrent iritis, ocular hypertension, pigmented keratic precipitates, iris atrophy, and corneal endothelial dysfunction. We report a 72-year-old immunocompetent man initially diagnosed with bilateral granulomatous uveitis of unknown origin, who was later confirmed by aqueous humor polymerase chain reaction to have CMV-AU. In retrospect, typical features of CMV-AU were present, but the bilateral involvement and atypical diffuse circumferential peripheral iris atrophy contributed to the delayed diagnosis. Corneal endothelial cell loss and trabecular meshwork depigmentation were also observed, consistent with reported CMV-related findings. The patient was treated with topical ganciclovir prepared from an injectable formulation, together with steroids and anti-glaucoma medications. While trabeculotomy provided some intraocular pressure (IOP) reduction, trabeculectomy proved more effective in achieving long-term IOP stability, consistent with previous reports. This case illustrates that CMV-AU may present bilaterally with atypical features and highlights the importance of early recognition, particularly for ophthalmology residents and general ophthalmologists who are not specialized in uveitis or glaucoma.

## Introduction

Uveitis is one of the major causes of blindness, with etiologies ranging from infectious to non-infectious. Among them, anterior uveitis is the most frequently encountered in clinical practice, and viral involvement has received increasing attention [1,2]. Traditionally, anterior uveitis caused by herpesviruses (HAU) has been mainly attributed to herpes simplex virus (HSV) and varicella-zoster virus (VZV) [2]. However, it has become evident that cytomegalovirus (CMV) can also be a causative agent [1-3]. CMV, a member of the herpesvirus family, typically does not cause noticeable pathology in immunocompetent individuals. In contrast, serious complications, such as infectious retinitis, have been reported in immunosuppressed individuals. In immunocompetent patients, ocular manifestations are usually confined to the anterior segment [4].

CMV-associated anterior uveitis (CMV-AU) may present either as recurrent hypertensive iritis resembling Posner-Schlossman syndrome or as chronic granulomatous anterior uveitis [3,4]. Differentiating HAU subtypes is essential for initiating specific antiviral therapy [1,5]. However, in general, the three major subtypes of HAU share overlapping clinical features, and thus, polymerase chain reaction (PCR) analysis of the aqueous humor is required for definitive viral identification [6]. Common features of HAU include mutton-fat keratic precipitates, often pigmented; iris atrophy, elevated intraocular pressure (IOP), and posterior synechiae with a pupil-pulling appearance distinct from other uveitides [6]. Importantly, HAU typically presents unilaterally, and iris atrophy is usually segmental or sectorial [1,3,4,6-10].

Herein, we report a case that was initially diagnosed as granulomatous uveitis of unknown origin and subsequently identified as CMV-AU three years later. In retrospect, several findings were suggestive of HAU from the outset; however, the bilateral involvement and the presence of a unique diffuse and circumferential peripheral iris atrophy likely contributed to the delayed diagnosis. Sharing this case may assist other clinicians in recognizing similar presentations in the future.

## Case presentation

In October 2021, a 72-year-old Japanese man presented to a local ophthalmology clinic with a corneal foreign body in his left eye (LE). At that time, bilateral iritis was incidentally noted, and topical steroid therapy was initiated. His medical history was notable for systemic hypertension and smoking, with no other significant systemic diseases.

In April 2022, he was referred to the Department of Ophthalmology at Shimane University Hospital for bilateral intraocular inflammation and elevated IOP, measuring 30 mmHg in the right eye (RE) and 26 mmHg in the LE. He had been prescribed 0.1% betamethasone eye drops four times daily and three classes of anti-glaucoma medications. FP receptor agonists had not been administered. At presentation, his best-corrected visual acuity (BCVA) was 0.6 in the RE and 0.4 in the LE, with IOPs of 16 mmHg RE and 14 mmHg LE measured with a Goldmann applanation tonometer (GAT). Slit-lamp examination revealed pigmented keratic precipitates (KPs) and mild vitreous opacities in both eyes (BE). Gonioscopy demonstrated open angles with no trabecular meshwork (TM) pigmentation in BE, with localized peripheral anterior synechiae (PAS) in the RE, but no angle nodules. No retinal hemorrhages or exudates were observed. The LE exhibited exotropia.

Laboratory investigations conducted at the initial visit to Shimane University Hospital showed urinalysis with a specific gravity of 1.017 and a pH of 6.5. Glucose, occult blood, and ketones were negative, while protein was slightly positive (± 15 mg/dL). Complete blood count (CBC) revealed mildly elevated white blood cells (9.46 × 10³/µL), slightly decreased red blood cells (4.25 × 10⁶/µL), and mildly increased mean corpuscular volume (MCV) and mean corpuscular hemoglobin (MCH). Hemoglobin (14.3 g/dL) was normal. Blood chemistry revealed mildly elevated blood urea nitrogen (BUN, 22.3 mg/dL) and fasting glucose (114 mg/dL), while glycated hemoglobin (HbA1c, NGSP) was 5.8%, within the normal range. Liver and renal function, electrolytes, and C-reactive protein (CRP) were normal. Immunology showed a low-titer positive antinuclear antibody (ANA, 1:40), whereas rheumatoid factor (RF), angiotensin-converting enzyme (ACE), and lysozyme were within normal ranges. Viral serology was positive for CMV IgG and VZV IgG, suggesting past infection; immunoglobulin M (IgM) antibodies were negative. Chest X-ray revealed no bilateral hilar lymphadenopathy (BHL). The head MRI showed only a small chronic cerebral infarction in the left pontine base. He was diagnosed with bilateral granulomatous uveitis and secondary glaucoma. Exotropia was considered unrelated, having been present for more than 10 years. Topical steroids and anti-glaucoma medications were continued.

Over the next three years at Shimane University Hospital, IOP fluctuated markedly (11.7-48 mmHg RE, 12.3-48 mmHg LE). In November 2024, BCVA decreased to 0.5 RE and 0.15 LE. The patient underwent cataract extraction combined with microhook trabeculotomy in the LE. Postoperatively, corneal edema developed in the LE, reducing BCVA to 0.02 and elevating IOP to 40 mmHg, prompting referral to the glaucoma clinic at Shimane University Hospital. At that time, he was using 0.1% betamethasone four times daily and four classes of anti-glaucoma medications.

**Figure 1:**
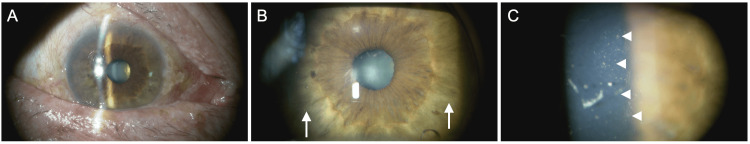
Anterior segment findings of the right eye at the initial visit to the glaucoma clinic Slit-lamp examination (A) and high-magnification images (B, C). Depigmentation of the peripheral iris is observed circumferentially (arrows). Numerous fine deposits are seen on the posterior surface of the cornea (arrowheads).

**Figure 2:**
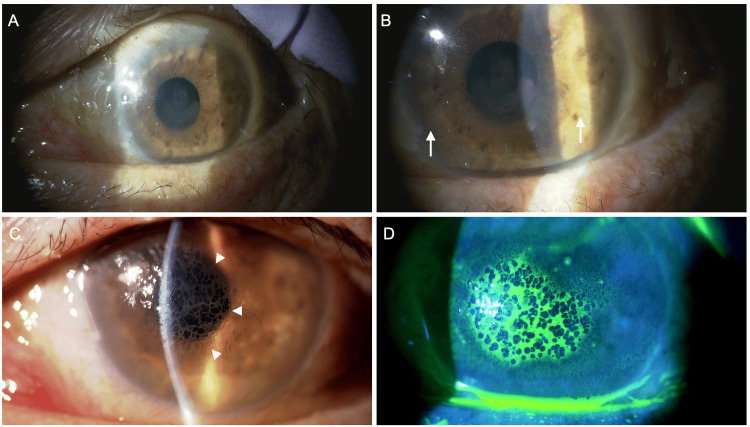
Anterior segment findings of the left eye at the initial visit to the glaucoma clinic Slit-lamp examination (A) and high-magnification images (B, C, D) Depigmentation of the peripheral iris is observed circumferentially (arrows). Lattice-like epithelial lesions are observed on the corneal surface (arrowheads). Fluorescein staining reveals that the lesion is localized to the corneal epithelium.

**Figure 3:**
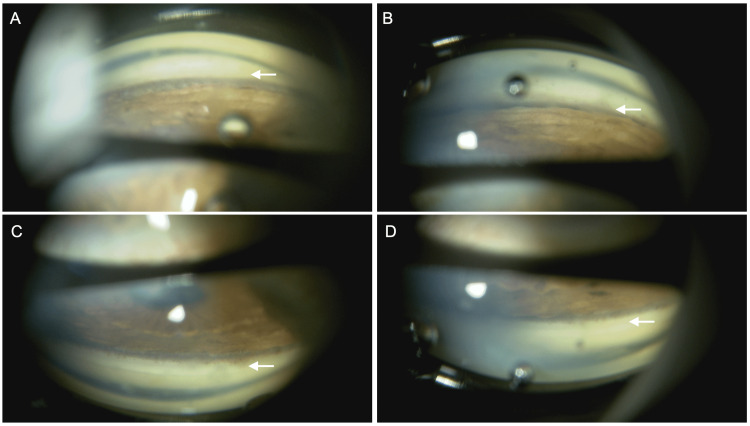
Gonioscopic findings of both eyes at the initial visit to the glaucoma clinic The angles of the inferior (A, B) and superior (C, D) quadrants of the right eye (A, C) and the left eye (B, D) are widely open. In both eyes, there is trace pigmentation of the trabecular meshwork (arrows).

**Figure 4:**
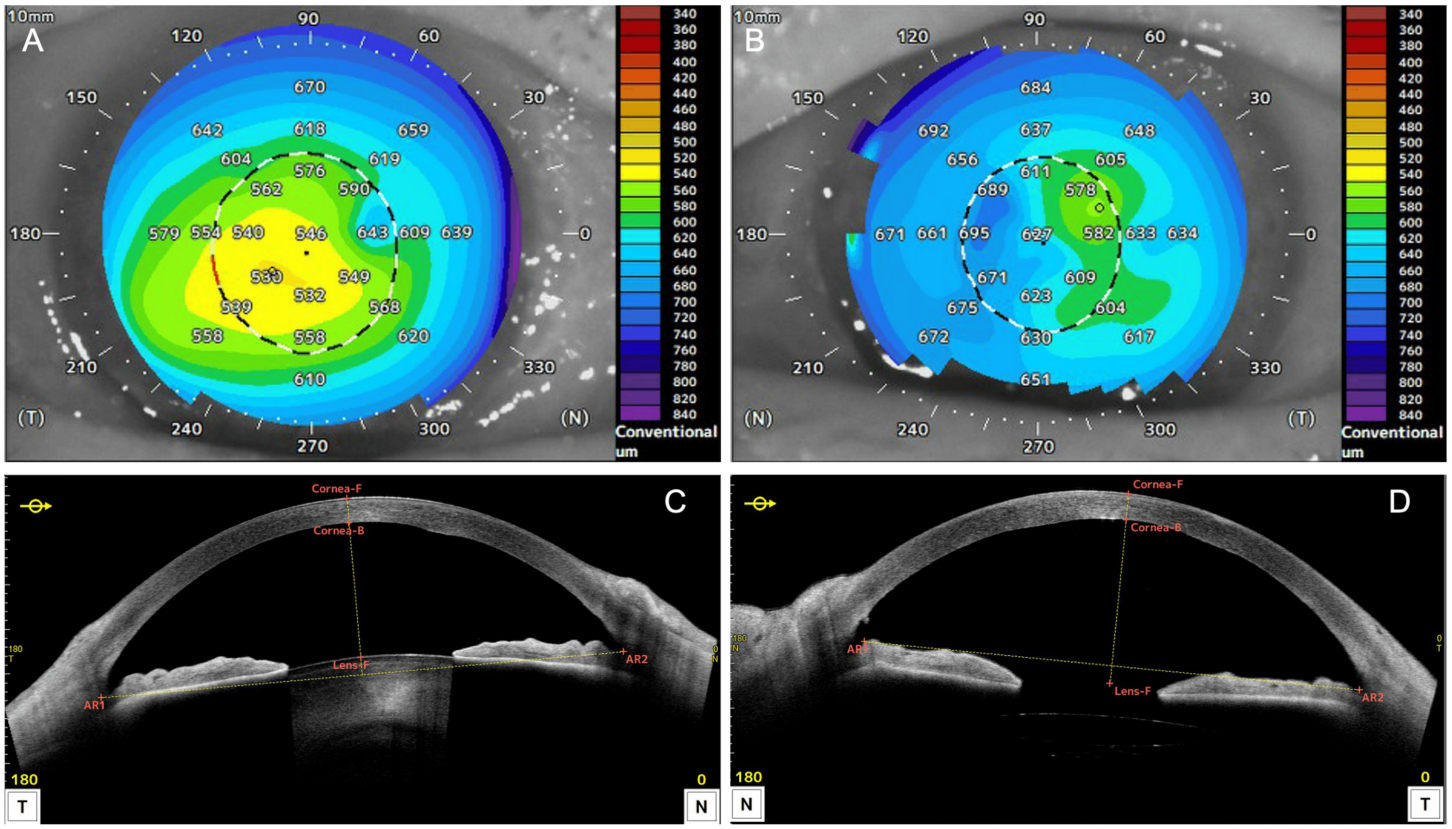
Corneal thickness map (A, B) and horizontal corneal cross-section (C, D) of the right (A, C) and left (B, D) eyes obtained using AS-OCT at the initial visit to the glaucoma clinic In both eyes, corneal thickening is prominent on the nasal side (A-D). T, temporal; N, nasal; F, front; B, back; AR, angle recess. AS-OCT, anterior segment optical coherence tomography

**Figure 5:**
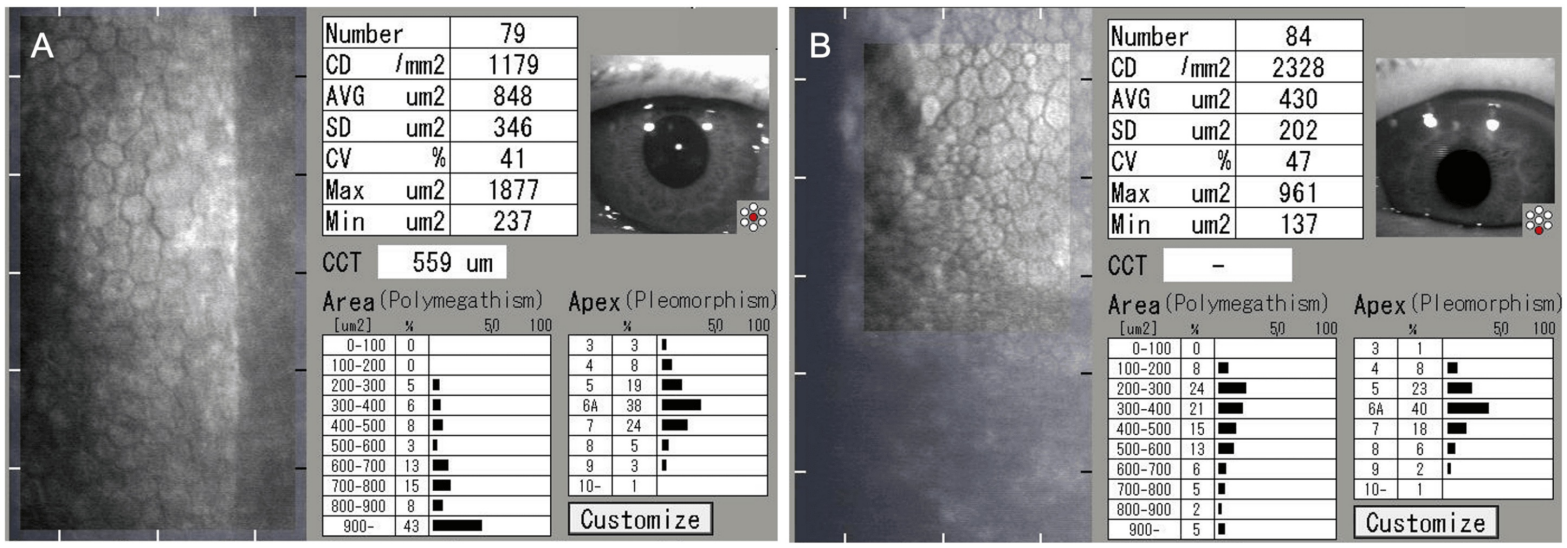
Specular microscopy findings of the right eye (A) and the left eye (B) at the initial visit to the glaucoma clinic Corneal endothelial cell density is 1179 cells/mm² in the right eye and 2328 cells/mm² in the left eye. CD, corneal endothelial cell density; AVG, average cell area; SD, standard deviation; CV, coefficient of variation; CCT, central corneal thickness

In December 2024, at the initial glaucoma clinic visit, BCVA was 0.15 LE, with IOPs of 25 mmHg RE and 33 mmHg LE (GAT). The LE exhibited a relative afferent pupillary defect (RAPD). Slit-lamp findings are shown in Figures 1, 2. The RE showed no conjunctival hyperemia (Figure 1A), no corneal edema, and no anterior chamber cells, but diffuse peripheral iris depigmentation (Figure 1B) and numerous brown KPs (Figure 1C). The LE also showed diffuse peripheral iris depigmentation (Figure 2B) with lattice-like epithelial lesions and stromal edema of the cornea (Figures 2C, 2D). A mild cataract was noted in the RE; intraocular lens fixation was stable in the LE. Gonioscopy revealed wide open angles in BE with only trace trabecular pigmentation, without PAS, angle nodules, or rubeosis (Figures 3A, 3B). A trabeculotomy cleft was observed nasally in the LE. The cup-to-disc ratio was 0.8 × 0.7 BE. No macular abnormalities, retinal vasculitis, exudates, or chorioretinal atrophy were observed. Anterior segment optical coherence tomography (OCT) (Casia 2, Tomey Corporation, Nagoya, Japan) revealed increased corneal thickness, particularly nasally in BE (Figure 4A-4D). Specular microscopy (EM-3000, Tomey Corporation) showed corneal endothelial cell density (CECD) of 1,179 cells/mm² RE and 2,328 cells/mm² LE (Figures 5A, 5B), with a marked reduction in the RE.

Given the characteristic peripheral iris depigmentation in BE, recurrent iridocyclitis, and a history of IOP spikes, viral infection was suspected. On the day of the glaucoma clinic visit, aqueous humor was aspirated from the LE for PCR testing. The results were positive for CMV and negative for HSV-1, HSV-2, VZV, EBV, and HHV-6. Treatment was initiated with 0.1% betamethasone three times daily, dorzolamide/timolol fixed combination twice daily, and 0.5% ganciclovir eye drops (prepared in-house from an injectable formulation) four times daily. Two weeks later, BCVA improved to 0.4 RE and 0.3 LE, with IOPs of 23 mmHg RE and 11 mmHg LE (non-contact tonometer, NCT). During follow-up, coin-shaped KP arrangements were observed in the RE.

**Figure 6:**
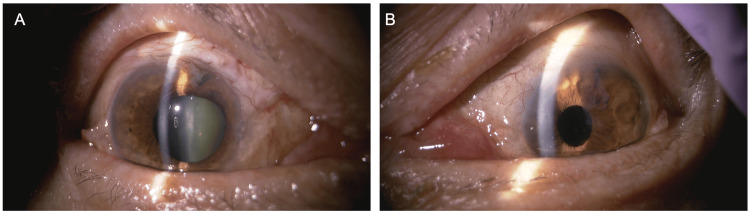
Anterior segment findings of both eyes at the final visit (nine months after the initial visit to the glaucoma clinic) In the right eye (A), keratic precipitates have disappeared, and the cornea is clear. In the left eye (B), the lattice-like corneal lesion has resolved, and the cornea is clear. In both eyes, circumferential depigmentation of the peripheral iris remains unchanged.

**Figure 7:**
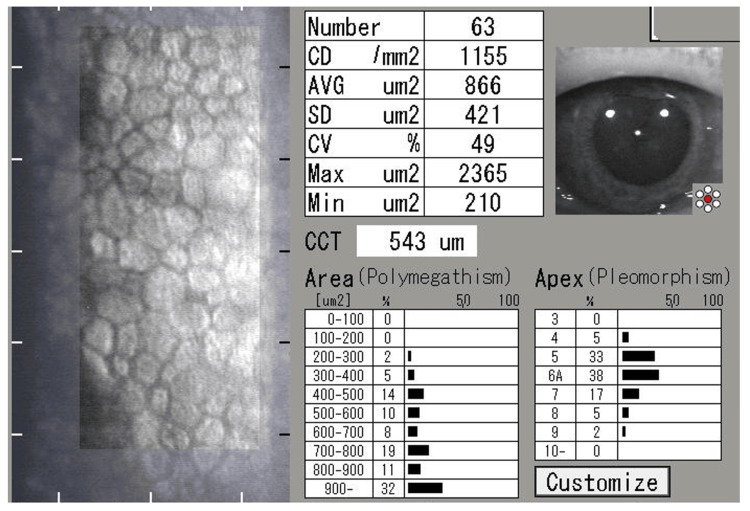
Specular microscopy finding of the right eye at the final visit (nine months after the initial visit to the glaucoma clinic) The corneal endothelial cell density is 1155 cells/mm², and the central corneal thickness is 543 µm, remaining unchanged from nine months earlier. Specular microscopy findings of the left eye are not recorded. CD, corneal endothelial cell density; AVG, average cell area; SD, standard deviation; CV, coefficient of variation; CCT, central corneal thickness

In June 2025, IOP increased to 45 mmHg RE (GAT), and the patient underwent trabeculectomy with mitomycin C and subconjunctival triamcinolone acetonide (20 mg) injection. Postoperatively, IOP decreased successfully. At the final follow-up in August 2025, BCVA was 0.3 BE, with IOPs of 9 mmHg RE and 12 mmHg LE (GAT). A functioning bleb was present in the RE, with no corneal edema or KPs in BE (Figures 6A, 6B). CECD in the RE was 1,155 cells/mm² (LE not assessed) (Figure 7). Humphrey Central 30-2 visual field testing (Carl Zeiss Meditec, Tokyo, Japan) showed mean deviations (MD) of -4.55 dB RE and -9.33 dB LE. The patient continued treatment with 0.1% betamethasone and 1% ganciclovir eye drops four times daily BE, along with dorzolamide/timolol and ripasudil eye drops twice daily in the LE.

## Discussion

CMV-AU can present with clinical features resembling Posner-Schlossman syndrome or Fuchs heterochromic iridocyclitis [8], which may complicate diagnosis. The diagnosis of HAU, including CMV-AU, is confirmed by PCR testing of the aqueous humor [6]. Therefore, suspecting HAU based on clinical findings is critical for establishing a definitive diagnosis. In retrospect, the characteristic features of CMV-AU--recurrent anterior uveitis, ocular hypertension, pigmented keratic precipitates, iris atrophy, and corneal endothelial dysfunction [4,6,9]--were all present in our patient. However, several atypical findings for HAU contributed to the delayed diagnosis.

Previous case series reported that all CMV-AU cases were unilateral [6,7,10]. In a study of 259 HAU patients, all HSV-AU and VZV-AU cases were unilateral, whereas only 3% of CMV-AU cases were bilateral [1]. The bilateral presentation in our case was therefore extremely atypical for HAU. Iris atrophy is a common feature across all HAU types. Typically, HAU is associated with iris atrophy described as segmental, sectorial, or patchy [3,6,10]. Although not widely recognized among general ophthalmologists, diffuse iris atrophy has been reported to occur relatively frequently in CMV-AU [1,10]. In our patient, circumferential peripheral iris atrophy was observed, while the pupillary area was spared. However, the etiology of the unique iris atrophy observed in this case remains unknown. It is possible that diseases other than CMV-AU, as well as genetic background or individual variability, may have contributed. Moreover, because the disease was bilateral, there was no interocular difference in iris color, making it difficult to associate iris atrophy with pathological significance. Corneal endothelial cell loss is an important finding suggestive of CMV infection [1,10]. In our case, marked asymmetry of CECD, with greater reduction in RE, was detected only after trabeculotomy LE. During the postoperative hypertensive phase, the LE also developed reticular epithelial lesions. Since both eyes exhibited corneal stromal thickening, CMV-associated endothelial damage was suspected. On gonioscopy, the angles were open without trabecular pigmentation. All three types of HAU may cause trabecular meshwork (TM) hyperpigmentation, but depigmentation of the TM is almost exclusively associated with CMV-AU [1], consistent with the angle findings in our case. Collectively, this case illustrates that CMV-AU may rarely present bilaterally, occasionally show diffuse circumferential iris atrophy, manifest corneal endothelial damage not readily detectable in the early course, and exhibit TM depigmentation. These features highlight its educational value, particularly for ophthalmology residents and general ophthalmologists not specialized in uveitis or glaucoma.

As a specific treatment for CMV-AU and CMV corneal endotheliitis, 0.15% ganciclovir gel has been approved in some countries. However, in regions where this formulation is not available, including Japan, hospital-prepared topical solutions made from diluted injectable formulations are used for treatment [5,11,12]. In experimental settings, 0.15% ganciclovir gel was reported to maintain therapeutic concentrations in the corneal endothelium for up to 12 h, exceeding the ED50 [11]. In contrast, ganciclovir solutions (0.5% and 1.0%) maintained effective levels above the ED50 for up to 6 h, with a rapid decline thereafter [11]. Therefore, we instructed the patient to instill the eye drops four times daily. Similar to previous reports [5], continued administration of ganciclovir and steroid eye drops in our case appeared to suppress both inflammation (i.e., KPs recurrence) and IOP elevation.

For CMV-related secondary glaucoma, trabeculectomy has been shown to provide superior IOP control compared with trabeculotomy [13]. Many eyes initially treated with trabeculotomy require subsequent trabeculectomy within a year, while trabeculectomy effectively stabilizes IOP and may facilitate discontinuation of antiviral therapy [13]. In our patient, postoperative ocular hypertension persisted LE after trabeculotomy until ganciclovir eye drops were initiated, and long-term triple antiglaucoma therapy remained necessary. In contrast, trabeculectomy RE achieved satisfactory IOP reduction, consistent with previously reported findings.

This is a single case report, and the diagnostic and therapeutic approaches described herein cannot be generalized. Nevertheless, analyzing and sharing the possible factors that delayed the definitive diagnosis of CMV-AU is valuable for clinical practice. This case demonstrates that CMV-AU may present with atypical features for HAU, highlighting the importance of awareness among clinicians.

## Conclusions

This case demonstrates that CMV-AU, although typically unilateral, can rarely present bilaterally with atypical features such as circumferential peripheral iris atrophy and trabecular meshwork depigmentation. The delayed recognition in this patient highlights the importance of considering CMV-AU even when findings are not classic for HAU. Early diagnosis by aqueous humor PCR and timely initiation of antiviral therapy are essential to preserve corneal endothelial function and prevent secondary glaucoma. In addition, the superior long-term IOP control achieved with trabeculectomy compared to trabeculotomy in this case is consistent with previous reports and suggests the need for appropriate surgical selection. Awareness of such atypical presentations is critical, particularly for ophthalmology residents and general ophthalmologists, to improve diagnostic accuracy and optimize patient outcomes.
